# Surfactant Protein B Precursor Inhibit the Progression of Resectable Non-small Cell Lung Cancer by Suppressing eIF4F-mediated Immune Evasion and Cancer Stemness

**DOI:** 10.7150/ijbs.134006

**Published:** 2026-04-23

**Authors:** Yongheng Wen, Hao Luo, Xintong Ji, Yushu Zheng, Liang Zhang, Dan Jian, Jing Chen, Hong Zhang, Nan-Nan Zhao, Hua Jin, Cheng-Xiong Xu, Ren-Tao Wang

**Affiliations:** 1 School of Medicine, Chongqing University, Chongqing, 400030, China.; 2 College of Pulmonary and Critical Care Medicine, Chinese PLA General Hospital, Beijing,100091, China.; 3 Cancer Center, Daping Hospital, Army Medical University, Chongqing, 400042, China.; 4 Xinkang Hospital, Taian, 271000, China.; 5 Department of Thoracic Surgery, Daping Hospital, Army Medical University, Chongqing, 400042, China.; 6 Department of Thoracic Surgery, The Third Affiliated Hospital of Chongqing Medical University, Chongqing, 401120, China.

**Keywords:** resectable NSCLC, pro-SFTPB, cancer stemness, immune evasion, eIF4F

## Abstract

Postoperative progression remains a major challenge in resectable non-small cell lung cancer (rNSCLC), but its factors and mechanisms are not fully understood. Here, we found that 34%-43% of rNSCLC patients have lower surfactant protein B precursor (pro-SFTPB) expression in tumor compared to adjacent tissues, and this low expression of pro-SFTPB is associated with low major pathological response to neoadjuvant chemoimmunotherapy and recurrence in rNSCLC. *In vitro* and animal experiments have shown that pro-SFTPB inhibits cancer stemness and immune evasion in NSCLC, and promotes the efficacy of PD-1 inhibitors. Targeting eIF4F improves the sensitivity of NSCLC with pro-SFTPB low expression to PD-1 inhibitors. Mechanistically, eIF4F promotes pro-SFTPB expression, while pro-SFTPB inhibits the formation of eIF4F complex by binding to eIF4A1, thereby suppressing the translation c-myc and PD-L1 that promote cancer stemness and immune evasion. Finally, we demonstrated that the reduction of SFTPB mRNA in NSCLC disrupts the negative regulation of pro-SFTPB on eIF4F complex formation, leading to activation of the eIF4F translation pathway and ultimately promoting tumor stemness and immune escape. In conclusion, the downregulation of pro-SFTPB in rNSCLC is an important factor in activating eIF4F-mediated cancer stemness and immune evasion to induce resistance to immunotherapy and promote disease progression.

## Introduction

Early-stage diagnosis and resection of non-small cell lung cancer (NSCLC) can improve patient survival. However, 30% to 75% of patients with resectable NSCLC (rNSCLC) experience postoperative recurrence, which is the main cause of death 1, 2. Recent clinical trials have shown that neoadjuvant PD-1 inhibitors plus platinum-based chemotherapy can improve the clinical outcomes of patients with rNSCLC 3-5. However, approximately 40% of rNSCLC patients who receive neoadjuvant PD-1 inhibitor plus chemotherapy still experience progression within 3 years after surgery 6. Unfortunately, the factors and mechanisms leading to postoperative progression in rNSCLC patients who received neoadjuvant PD-1 inhibitors plus chemotherapy are unclear.

Accumulating evidence indicates that increased immune evasion and cancer stemness play critical roles in cancer progression and resistance to immunotherapy and chemotherapy 7, 8, and dysregulated mRNA translation is an important feature of cancer immune evasion 9 and cancer stem cells 10. Eukaryotic initiation factor 4F (eIF4F) is a translation initiation factor composed of eIF4A, eIF4E, and eIF4G. Studies have shown that eIF4F-mediated mRNA translation is dysregulated in cancers and that it promotes a specific malignant phenotype through the selective translation of 'eIF4F-sensitive oncogene mRNAs' 11-15. For example, Cerezo et al. reported that activation of the eIF4F complex upregulates PD-L1 expression by increasing the translation of STAT1 mRNA 14, and Lin et al. reported that eIF4F upregulates the translation of c-myc mRNA 15. Importantly, upregulated PD-L1^14^, ^16^, ^17^ and c-myc expression 18-20 in cancers are closely associated with immune evasion, cancer stemness, and resistance to immunotherapy and chemotherapy. Collectively, these findings suggest that activation of the eIF4F pathway in cancer may lead to simultaneous activation of cancer stemness and immune evasion. However, there are currently no reports indicating that activation of the eIF4F pathway in cancers can simultaneously promote immune evasion and cancer stemness. In addition, the molecular mechanism underlying the abnormal formation of the eIF4F complex in NSCLC is not yet fully understood.

Pro-SFTPB is a precursor of pulmonary surfactant protein B (SFTPB), and its abnormally low expression is associated with several pulmonary diseases 21, including NSCLC 22, 23. The SFTPB gene is first transcribed/translated into a monomeric protein of approximately 40-kDa, and then modified by glycosylation and signal peptide cleavage resulting in the 42-kDa pro-SFTPB within the endoplasmic reticulum 24, 25. In a previous study, we demonstrated that low expression of pro-SFTPB promotes early-stage NSCLC progression by activating the Akt signaling pathway in a nude mouse xenograft model 22. However, in a subsequent study, inhibition of the Akt pathway with an Akt inhibitor in a C57BL/6J mouse xenograft model without immune deficiency did not significantly inhibit the growth of lung cancer with low pro-SFTPB expression (Fig. S1). In addition, Pocha et al. reported that lung adenocarcinoma brain metastases with low expression of surfactants exhibit cold tumor characteristics 23. These findings suggest that low expression of pro-SFTPB may promote the progression of NSCLC by regulating the cancer immune response, but the underlying molecular mechanism is unclear.

In this study, we found that the eIF4F complex stimulates pro-SFTPB protein expression, while pro-SFTPB protein inhibits the formation of eIF4F complex by binding to eIF4A1, thereby constituting a negative feedback loop between eIF4F and pro-SFTPB to regulate the initiation of eIF4F-mediated mRNA translation. In rNSCLC, the decrease in SFTPB mRNA levels caused by smoking leads to low expression of pro-SFTPB protein, resulting in abnormal formation of eIF4F complex, which upregulates the expression of eIF4F downstream genes such as PD-L1 and c-myc that promote cancer stemness and immune escape, ultimately leading to chemoimmunotherapy resistance and recurrence in rNSCLC. Notably, targeting eIF4F can enhance the therapeutic efficacy of PD-1 inhibitors in NSCLC with low expression of pro-SFTPB.

## Materials and Methods

### Cell lines and human samples

Human pulmonary alveolar epithelial cell (HPAEpiC) and Lewis lung cancer (LLC) cells were cultured in Dulbecco`s modified Eagle`s medium (Sperikon Life Science & Biotechnology co., Ltd, Deyang, China) supplemented with 10% fetal bovine serum (FBS) (Jiangsu Aidisheng Biological Technology Co., Ltd, Yancheng, China). H1299, H460, PC-9 and H1650 cells were cultured in RPMI1640 medium (Sperikon Life Science & Biotechnology co., Ltd) with 10% FBS.

NSCLC specimens were collected from resectable lung adenocarcinoma (LUAD) patients (Table S1 and Table 1) during surgery at Chinese PLA General Hospital under a protocol approved by the ethical review committees (S-2025-011-01). PD-L1 positive (TC >1%) stage IIIA LUAD patients received pembrolizumab combined with platinum and pemetrexed chemotherapy every 3 weeks. After 2 cycles of neoadjuvant therapy stage IIIA LUAD patients underwent surgery.

### CD8^+^ T cell migration assay and co-culture of CD8^+^ T cells and NSCLC cells

For the CD8^+^ T cell migration assay, indicated NSCLC cells were transfected with the indicated plasmids, and the cell culture medium was changed after 48 hours of transfection. After another 48 hours of culture, the supernatant was transferred into the lower chamber of the transwell (Corning Inc, Athol, MA, USA) and 5×10^5^ activated CD8^+^ T cells in 100μL medium that had been incubated with anti-human CD8α APC-Cy7 antibody (Invitrogen, Carlsbad, CA, USA) were added into the upper chamber of transwell. After 3 hours, the migrated cells were detected by flow cytometry.

To measure the cytotoxicity of CD8^+^ T cells, indicated NSCLC cells were transfected with indicated plasmids for 72 hours and then cocultured with activated CD8^+^ T cells at a ratio 1:10. After coculturing for 12 hours, dead tumor cells were detected using flow cytometry analysis and the concentration of interferon-γ in culture medium was measured using Elisa kit (Abcam, Boston, USA). The plasmids expressing pro-SFTPB or eIF4A1 were purchased from Genecreate (Wuhan, China) and iGeneBio (Guangzhou, China), respectively. The target sequences for pro-SFTPB and eIF4A1 were provided in Table S2.

### Immunohistochemistry (IHC), immunofluorescence (IF), co-immunoprecipitation (Co-IP), and western Blotting (WB)

Indicated cells were transfected with indicated plasmids for 72 hours, and then subjected to analysis. For 4EGI-1 treatment experiments, cells transfected for 48 hours were treated with 5umol/L 4EGI-1 for 24 hours, then subjected to WB. For CSE (cigarette smoke extract) treatment, CSE was extracted from cigarettes as described previously 26, and cells were treated with 10% CSE for 3 days, and the culture medium with 10% CES was changed every day. 4EGI-1 IHC, IF, Co-IP, and WB were performed as described previously 27. Pro-SFTPB, PD-L1, CD133, β-actin, and Flag antibodies were purchased from Proteintech (Wuhan, China); eIF4A1, ALDH1 and CD8 antibodies were purchased from Abcam; eIF4G and eIF4E antibodies were purchased from Bioss (Beijing, China).

### Sphere formation assay

Indicated NSCLC cells transfected with indicated plasmids for 48 hours, then subjected to sphere formation assay. The sphere formation assay was performed as described previously 28.

### RNA analysis and proteomics analysis

Total RNAs were extracted from tumor tissues or NSCLC cells using Trizol reagent (Beyotime Biotechnology, Shanghai, China). Reverse transcription and polymerase chain reaction were performed with PrimeScript^TM^ RT Master Mix (Takara, Japan) and Taq qPCR mix (GeneCopoeia Inc, Rockville, MD, USA), respectively. The primer sequences for genes were defined as follows: SFTPB forward, 5'-CACCTCATCCTTGGCCTGTG-3'; reverse, 5'-CTTGGCATAGGTCATCGGCTC-3'; β-actin forward, 5'-CGTCACCAACTGGGACGA-3'; reverse, 5'-ATGGGGGAGGGCATACC-3'. For proteomics analysis, H1299 cells were transfected with plasmids that expressing scramble or shRNA of pro-SFTPB for 72 hours, then proteins were extracted. Proteomics analysis was performed as described previously 28.

### Animal experiments

Indicated cells were transfected with indicated plasmids for 72 hours, then, subjected to animal model construction. 6-weeks-old female nude mice and C57BL/6J mice (Beijing HFK Bioscience, Beijing, China) were used for animal experiments. To investigate the effects of pro-SFTPB on the tumorigenicity of NSCLC cells, indicated numbers of NSCLC cells were transplanted subcutaneously on the backs of nude mice. To investigate the effects of pro-SFTPB on tumor immune evasion, 1×10^6^ LLC cells were transplanted subcutaneously on the backs of C57BL/6J mice. The animal experiments on tumor formation and immune evasion were conducted for one month. To investigate the therapeutic effect of the combination of 4EGI-1 and PD-1/PD-L1 inhibitors on lung cancer with low expression of pro-SFTPB, a C57BL/6J xenograft model was constructed using pro-SFTPB knockdown LLC cells as described above. When tumor volumes reached about 50 mm^3^, mice were randomly divided into four groups. Control group mice were treated with PD-1 mAb alone (1 mg/Kg body weight, every three days) (Pembrolizumab, Merk & Co., Inc., Kenilworth, NJ, USA), other groups were treated with PD-1 mAb and/or 4EGI-1 (75 mg/Kg body weight, i.p. 5days a week). The tumor volume was measured every three days.

### Statistical analysis

All data were presented as the mean ± standard deviation and statistical differences were analyzed using SPSS software. Differences were considered statistically significant at *p*-value of less than 0.05. The survival was calculated by Kaplan-Meier survival analysis.

## Results

### Low expression of pro-SFTPB in tumors compared with that in adjacent tissues is related to a decreased immune response and increased cancer stemness in early-stage NSCLC

**Figure 1:**
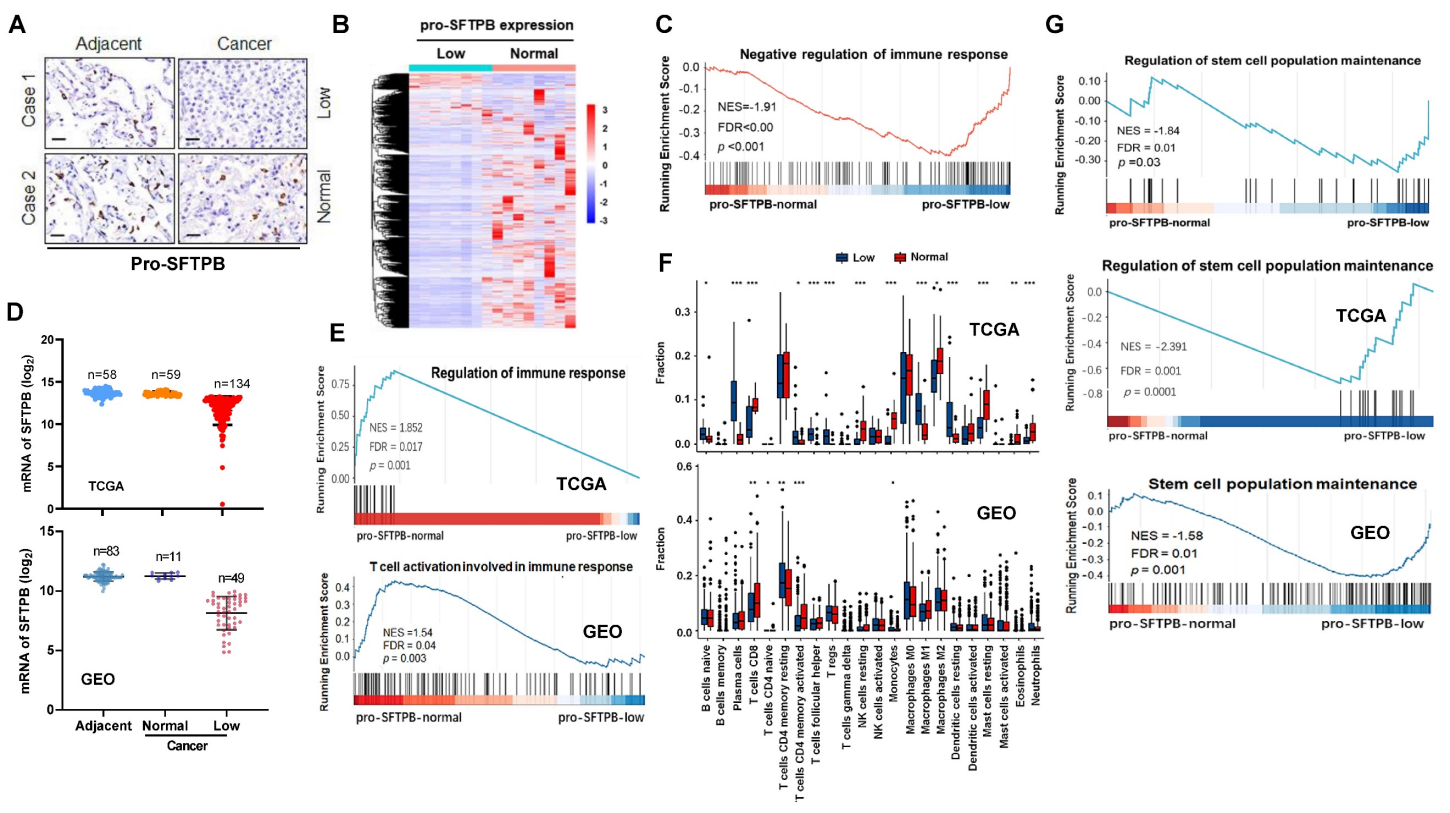
** Bioinformatics analysis shows that low expression of pro-SFTPB in tumors compared with that in adjacent tissues is related to decreased immune response and increased cancer stemness in early-stage non-small cell lung cancer (eNSCLC). (A)** Immunohistochemistry images of pro-SFTPB in eNSCLC and their corresponding adjacent tissues.** (B)** Heatmap of differentially expressed genes between eNSCLC with low expression (n=8) and normal expression of pro-SFTPB (n=8). **(C)** The correlation between pro-SFTPB low expression and immune response in eNSCLC was investigated by Gene Set Enrichment Analysis (GSEA) of RNA sequencing data. **(D)** According to the comparison of SFTPB mRNA expression levels between tumor tissue and adjacent tissue, eNSCLC patients were divided into pro-SFTPB normal expression group and low expression group. Data from TCGA and GEO (GSE75037) database. **(E)** The correlation between pro-SFTPB low expression and immune response in eNSCLC was investigated by GSEA of TCGA and GEO (GSE75037) datasets. **(F)** The ratio differentation of immune cells between eNSCLC with low and normal pro-SFTPB expression was investigated by CIBERSORT analysis of the TCGA and GEO datasets, and Wilcoxon rank sum was used for the significance test.** (G)** The correlation between pro-SFTPB low expression and cancer stem cell population maintenance in eNSCLC was investigated by GSEA of PLA hospital clinical sample RNA sequencing data as well as the TCGA and GEO (GSE75037) datasets.

First, we investigated whether low expression of pro-SFTPB is involved in the immune response in early-stage NSCLC. On the basis of the immunohistochemistry (IHC) results (Fig. 1A), early-stage NSCLC tissues with no difference in pro-SFTPB expression between tumors and adjacent tissues were placed in the normal pro-SFTPB expression group, whereas NSCLC tissues with lower pro-SFTPB expression in tumors than in adjacent tissues were placed in the low pro-SFTPB expression group, and RNA sequencing was performed on these tumors (Fig. 1B). Bioinformatics analysis was subsequently performed on the RNA sequencing data. Gene Ontology (GO) (Fig. S2A) and Gen Set Enrichment Analysis (GSEA) (Fig. 1C) revealed that low expression of pro-SFTPB is associated with a weak immune response in early-stage NSCLC. These results were further confirmed by analyzing RNA sequencing datasets of early-stage NSCLC in the TCGA and GEO databases (Fig. 1D, E, and Fig. S2B and S2C). Additionally, the CIBERSORT analysis results for both the TCGA and GEO datasets indicated that early-stage NSCLC patients with low expression of pro-SFTPB had a relatively low proportion of CD8^+^ T cells (Fig. 1F). Interestingly, bioinformatics analysis of our clinical cohort and datasets from the TCGA and GEO databases revealed that low expression of pro-SFTPB was positively correlated with cancer stem cell population maintenance (Fig. 1G and Fig. S2). Together, these data indicate that low expression of pro-SFTPB in tumors compared with that in adjacent tissues is closely correlated with a decreased immune response and increased cancer stemness in early-stage NSCLC.

### Downregulation of pro-SFTPB expression inhibits the CD8^+^ T-cell-mediated immune response in NSCLC

**Figure 2:**
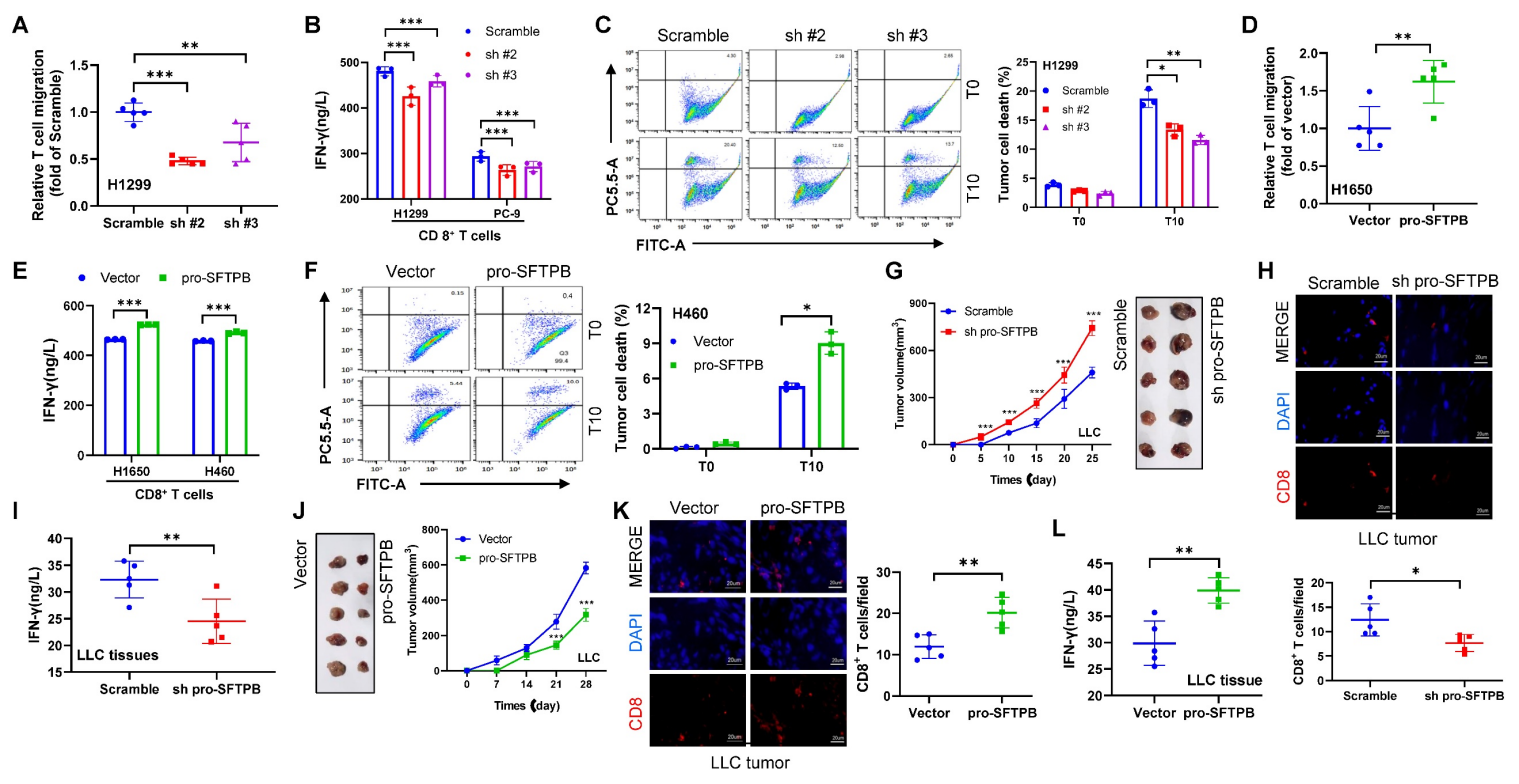
** pro-SFTPB positively regulates CD8^+^ T-cell-mediated immune response in NSCLC. (A)** The migration of CD8^+^ T cells incubated in the culture medium supernatant of H1299 cells transfected with plasmids expressing scramble or shRNA of pro-SFTPB was measured by transwell analysis.** (B)** The concentration of interferon-γ (IFN-γ) in the coculture medium of CD8^+^ T cells and NSCLC cells transfected with plasmids expressing scramble or shRNA of pro-SFTPB.** (C)** The death of H1299 cells transfected with plasmids expressing scramble or pro-SFTPB shRNA induced by CD8^+^ T cells was detected using flow cytometry.** (D)** The migration of CD8^+^ T cells incubated in the culture medium supernatant of H1650 cells transfected with vector or plasmids expressing pro-SFTPB was measured by transwell analysis. **(E)** The concentration of IFN-γ in the coculture medium of CD8^+^ T cells and NSCLC cells transfected with vector or plasmids expressing pro-SFTPB.** (F)** The death of H460 cells transfected with vector or plasmids expressing pro-SFTPB induced by CD8^+^ T cells was detected using flow cytometry. **(G)** Tumor images and growth curve in C57BL/6J xenograft models constructed using Lewis lung cancer (LLC) cells transfected with plasmids expressing scramble or shRNA of pro-SFTPB.** (H)** Detection of CD8^+^ T cells in LLC tumors by immunofluorescence (IF) (Tumors from G). **(I)** The concentration of IFN-γ in LLC tumors (Tumors from G). **(J)** Tumor images and growth curve in C57BL/6J xenograft models constructed using LLC cells transfected with vector or pro-SFTPB expressing plasmids. **(K)** Detection of CD8^+^ T cells in LLC tumors by IF (Tumors from J). **(L)** The concentration of IFN-γ in LLC tumors (Tumors from J). Scale bar=20 μm; T0, the ratio of tumor cells to T cells is 1:0; T10, the ratio of tumor cells to T cells is 1:10; sh pro-SFTPB, pro-SFTPB shRNA; sh#2, pro-SFTPB shRNA #2; sh#3, pro-SFTPB shRNA #3; *, *p* <0.05; **, *p* <0.01; ***, *p* <0.001.

To determine whether low expression of pro-SFTPB is directly involved in the CD8^+^ T-cell mediated immune response in NSCLC, we conducted a coculture of CD8^+^ T cells and NSCLC cells. For pro-SFTPB knockdown experiments, we selected H1299 and PC-9 NSCLC cells with pro-SFTPB expression levels similar to those of the human pulmonary alveolar epithelial cell line HPAEpiC, whereas H1650 and H460 cells with pro-SFTPB expression lower than that of HPAEpiC were selected for pro-SFTPB overexpression experiments (Fig. S3A, S3B and S3C). Our results revealed that coculturing pro-SFTPB-knockdown NSCLC cells with CD8^+^ T cells inhibited CD8^+^ T-cell migration (Fig. 2A) and IFN-γ secretion (Fig. 2B), as well as the ability of CD8^+^ T cells to kill tumor cells (Fig. 2C). In contrast, coculture with pro-SFTPB overexpressing NSCLC cells promoted CD8^+^ T-cell migration (Fig. 2D) and IFN-γ secretion (Fig. 2E), as well as the ability of CD8^+^ T cells to kill tumor cells (Fig. 2F). The *in vitro* results were further confirmed in animal models. The results of animal experiments revealed that knockdown of pro-SFTPB expression significantly promoted tumor growth and shortened the time to tumor formation in Lewis lung cancer (LLC) cells in C57BL/6J mice without immune deficiency (Fig. 2G). Additionally, significantly decreased CD8^+^ T-cell infiltration (Fig. 2H) and IFN-γ expression (Fig. 2I) were observed in LLC tumors with low expression of pro-SFTPB. In contrast, the overexpression of pro-SFTPB in LLC cells significantly inhibited tumor growth and delayed tumor formation time in C57BL/6J mice (Fig. 2J). Additionally, overexpression of pro-SFTPB significantly increased CD8^+^ T-cell infiltration (Fig. 2K) and IFN-γ expression (Fig. 2L) in LLC xenograft tumors. These findings indicate that pro-SFTPB positively regulates the CD8^+^ T-cell-mediated immune response in NSCLC.

### Downregulation of pro-SFTPB promotes cancer stemness in NSCLC

**Figure 3:**
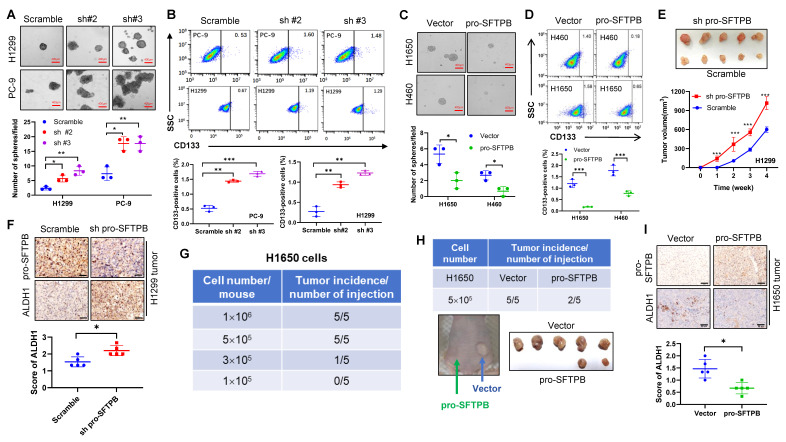
** pro-SFTPB negatively regulates NSCLC stemness. (A)** Sphere formation assay using pro-SFTPB-knockdown NSCLC cells and control cells. **(B)** Detection of CD133 positive cells by flow cytometry in pro-SFTPB-knockdown NSCLC cells and control cells. **(C)** Sphere formation assay using pro-SFTPB overexpressing NSCLC cells and control cells. **(D)** Detection of CD133 positive cells by flow cytometry in pro-SFTPB overexpressing NSCLC cells and control cells. **(E)** Tumor growth curve and tumor images in nude mouse xenograft models constructed using H1299 cells with or without knockdown of pro-SFTPB. **(F)** Immunohistochemistry (IHC) analysis of ALDH1 and pro-SFTPB in H1299 xenograft tumors (Tumors from E). **(G)** Tumor formation rate within one month after implantation of indicated numbers of H1650 cells into each nude mouse (n=5). **(H)** Tumor formation rate and tumor images in nude mouse xenograft models constructed using H1650 cells (5×10^5^ cells/mouse) transfected with pro-SFTPB expressing plasmid or vector (n=5). **(I)** IHC analysis of ALDH1 and pro-SFTPB in H1650 xenograft tumors (Tumors from H). All animal experiments were conducted for one month after implantation of NSCLC cells into nude mice. Scale bar=50 μm (F and I); Scale bar=400 μm (A and C); sh pro-SFTPB, pro-SFTPB shRNA; sh#2, pro-SFTPB shRNA #2; sh#3, pro-SFTPB shRNA #3; *, *p* <0.05; **, *p* <0.01; ***, *p* <0.001.

Next, we investigate whether low expression of pro-SFTPB is directly involved in cancer stemness regulation. Our results revealed that the knockdown of pro-SFTPB expression significantly increased the sphere formation ability of NSCLC cells (Fig. 3A) and the proportion of CD133-positive cells among NSCLC cells (Fig. 3B). In contrast, overexpression of pro-SFTPB significantly decreased the sphere formation ability of NSCLC cells (Fig. 3C) and the proportion of CD133-positive cells among NSCLC cells (Fig. 3D). The *in vitro* results were further confirmed in a nude mouse xenograft model. As shown in Fig. 3E, the knockdown of pro-SFTPB shortened the tumor formation time of H1299 cells in nude mice and promoted tumor growth. In addition, IHC analysis of H1299 xenograft tumors revealed that pro-SFTPB downregulation significantly upregulated the expression of the cancer stem cell marker protein ALDH1 (Fig. 3F). Importantly, when 5×10^5^ H1650 cells were implanted into each nude mouse, the tumor formation rate was 100% within one month (Fig. 3G), but overexpression of pro-SFTPB reduced the tumor formation rate of H1650 cells to 40% (Fig. 3H). Additionally, overexpression of pro-SFTPB significantly downregulated the expression of ALDH1 in H1650 xenograft tumors (Fig. 3I). These results indicate that pro-SFTPB negatively regulates cancer stemness in NSCLC.

### Downregulation of pro-SFTPB expression activates immune evasion and cancer stemness by increasing eIF4A1-mediated expression of c-myc and PD-L1 in NSCLC cells

**Figure 4:**
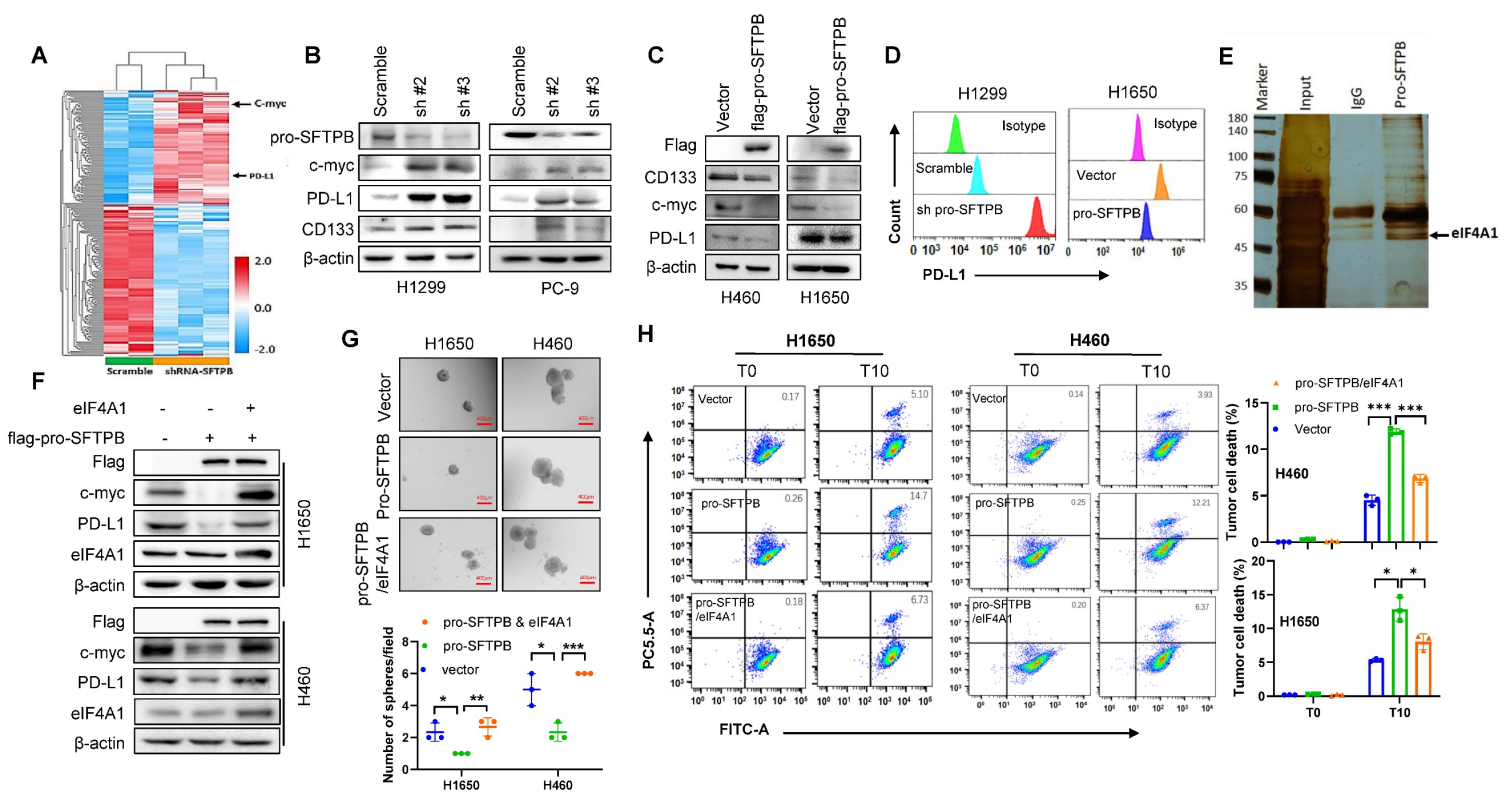
** pro-SFTPB inhibits immune evasion, cancer stemness, and the expression of c-myc and PD-L1 through inhibiting eIF4A1. (A)** Heatmap of differentially expressed proteins between pro-SFTPB-knockdown H1299 cells and control cells. **(B-C)** Western blot (WB) analysis showed pro-SFTPB negatively regulates the expression of c-myc, PD-L1 and CD133 in NSCLC cells. **(D)** Flow cytometry analysis showed pro-SFTPB negatively regulates PD-L1 expression in NSCLC cells. **(E)** Silver staining shows eIF4A1 interact with pro-SFTPB.** (F)** WB analysis shows that overexpression of eIF4A1 weakened the inhibitory effect of pro-SFTPB on the expression of c-myc and PD-L1 in NSCLC cells. **(G)** Overexpression of eIF4A1 weakened the inhibitory effect of pro-SFTPB on the sphere formation of NSCLC cells. Scale bar=400 μm. **(H)** Flow cytometry analysis shows that overexpression of eIF4A1 weakened the promoting effect of pro-SFTPB on CD8^+^ T cell cytotoxicity. WCL, whole cell lysate; sh#2, pro-SFTPB shRNA #2; sh#3, pro-SFTPB shRNA #3; *, *p* <0.05; **, *p* <0.01; ***, *p* <0.001.

To investigate the molecular mechanism through which low expression of pro-SFTPB promotes immune evasion and cancer stemness, we conducted proteomics analysis and observed that the expression of c-myc and PD-L1 was upregulated in pro-SFTPB-knockdown H1299 cells compared with control H1299 cells (Fig. 4A). Western blot analysis also revealed that the knockdown of pro-SFTPB increased the expression of c-myc, PD-L1 and the cancer stemness marker protein CD133 in NSCLC cells (Fig. 4B), whereas the overexpression of pro-SFTPB decreased the expression of c-myc, PD-L1 and CD133 in NSCLC cells (Fig. 4C). Furthermore, the negative regulatory effect of pro-SFTPB on PD-L1 expression in NSCLC cells was confirmed by cytometric analysis (Fig. 4D). To investigate the mechanism by which pro-SFTPB regulates the expression of c-myc and PD-L1 in NSCLC cells, we performed Co-IP/MS analysis. Because a previous study revealed that pro-SFTBP plays its role through binding with other proteins 22. Co-IP/MS analysis revealed that multiple proteins bind to pro-SFTPB (Fig. 4E). Among them, we chose eIF4A1 for subsequent experiments. Our data (Fig. S4A) and those of previous reports show that eIF4A1 increases the expression of PD-L1 29 and c-myc 30. Importantly, overexpression of eIF4A1 inhibited the overexpression of pro-SFTPB-induced downregulation of c-myc and PD-L1 expression in NSCLC cells (Fig. 4F). In addition, overexpression of eIF4A1 suppressed the inhibitory effects of pro-SFTPB on the sphere formation of NSCLC cells (Fig. 4G) and the stimulatory effects of pro-SFTPB on the tumor cell-killing ability of CD8^+^ T cells (Fig. 4H). Together, these findings indicate that pro-SFTPB suppresses immune evasion, cancer stemness, and PD-L1 and c-myc expression by inhibiting eIF4A1 in NSCLC.

### Pro-SFTPB inhibits c-myc and PD-L1 expression through blocking the assembly of the eIF4F complex by binding to eIF4A1

**Figure 5:**
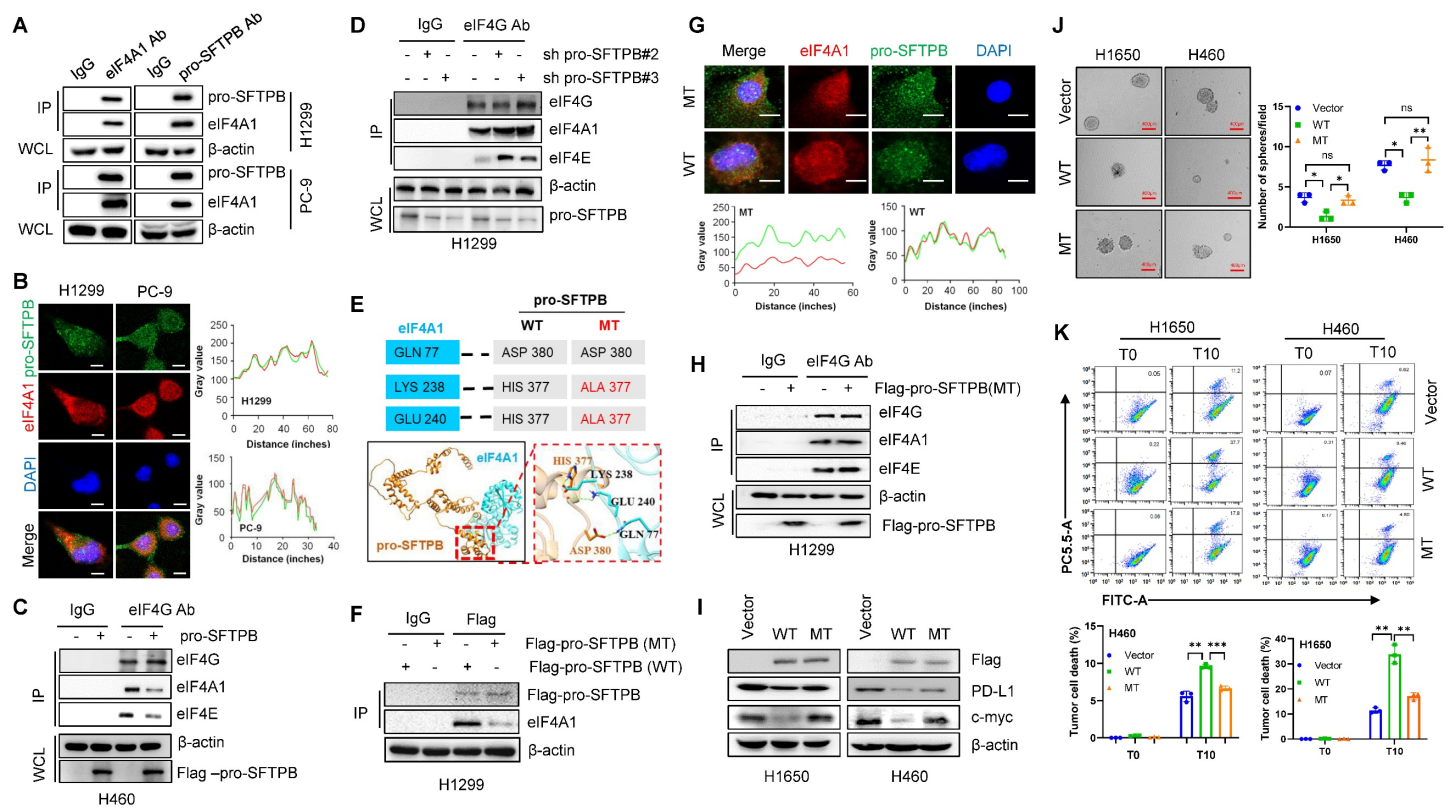
** pro-SFTPB inhibits immune evasion and cancer stemness in NSCLC by blocking the eIF4F complex formation through binding to eIF4A1. (A)** The interaction between eIF4A1 and pro-SFTPB was detected by Co-immunoprecipitation (Co-IP) in NSCLC cells. **(B)** Co-localization of pro-SFTPB and eIF4A1 in NSCLC cells was detected by immunofluorescence (IF). **(C)** Co-IP analysis showed overexpression of pro-SFTPB reduce the formation of eIF4F complex in H460 cells. **(D)** Co-IP analysis showed knockdown of pro-SFTPB increase the formation of eIF4F complex in H1299 cells. **(E)** The binding residues between pro-SFTPB and eIF4A1 was predicted using AlphaFold 3. **(F)** The effect of the pro-SFTPB mutation on the interaction between pro-SFTPB and eIF4A1 in H1299 cells was examined by Co-IP. **(G)** The effect of the pro-SFTPB mutation on the interaction between pro-SFTPB and eIF4A1 in PC-9 cells was examined by IF analysis. **(H)** The effect of the pro-SFTPB mutation on the eIF4F complex formation in H1299 cells was examined by Co-IP. **(I)** The effect of pro-SFTPB mutation on the expression of PD-L1 and c-myc in NSCLC cells were measured by Western blot. **(J)** The effect of the pro-SFTPB mutation on the sphere formation of NSCLC cells was measured. **(K)** The effect of pro-SFTPB mutation on the CD8^+^ T cell cytotoxicity was measured by flow cytometry after coculture of CD8^+^ T cells and NSCLC cells transfected plasmids expressing wild type or mutant pro-SFTPB. The gray value was measured by Image J. Scale bar=10 μm (B and G); Scale bar=400 μm (J); WT, wild type of pro-SFTPB; MT, mutant type of pro-SFTPB; *, *p* <0.05; **, *p* <0.01; ***, *p* <0.001.

To investigate how pro-SFTPB inhibits the effect of eIF4A1, we evaluated the effect of pro-SFTPB on eIF4A1 expression and found that pro-SFTPB did not affect eIF4A1 expression in NSCLC cells (Fig. S4B and S4C). Thus, we next investigated the effect of pro-SFTPB on the formation of the eIF4F complex. MS/Co-IP (Fig. 4E), Co-IP (Fig. 5A and B), and IF (Fig. 5B) analysis revealed that pro-SFTPB binds to eIF4A1 and that eIF4A1 is a core member of the translation initiation factor eIF4F. GEO dataset analysis revealed that pro-SFTPB is involved in the regulation of translation initiation (Fig. S2C). Our Co-IP analysis revealed that overexpression of pro-SFTPB reduced eIF4F complex formation (Fig. 5C), whereas knockdown of pro-SFTPB increased eIF4F formation in NSCLC cells (Fig. 5D), indicating that pro-SFTPB blocks eIF4F complex formation. To investigate whether pro-SFTPB inhibits the formation of the eIF4F complex by binding to eIF4A1, we predicted the binding sites between pro-SFTPB and eIF4A1 by AlphaFold 3 (https://www.alpha3manufacturing.com) and subsequently constructed a plasmid expressing pro-SFTPB with predicted binding site mutations (Fig. 5E). Co-IP (Fig. 5F) and IF analyses (Fig. 5G) revealed that changing the HIS377 of pro-SFTPB to ALA377 dramatically reduced the binding between pro-SFTPB and eIF4A1 in NSCLC cells. Importantly, overexpression of mutant pro-SFTPB did not affect the formation of the eIF4F complex (Fig. 5H). In addition, mutation of pro-SFTPB weakened the inhibitory effects of pro-SFTPB on the expression of c-myc and PD-L1 (Fig. 5I) and on sphere formation (Fig. 5J). Mutation also weakened the promoting effect of pro-SFTPB on the killing of tumor cells by CD8^+^ T cells (Fig. 5K). These findings indicate that pro-SFTPB inhibits the expression of c-myc and PD-L1 and suppresses immune evasion and cancer stemness by blocking eIF4F complex formation through binding to eIF4A1.

### Downregulation of pro-SFTPB expression leads to resistance to PD-1 inhibitors in lung cancer

**Figure 6:**
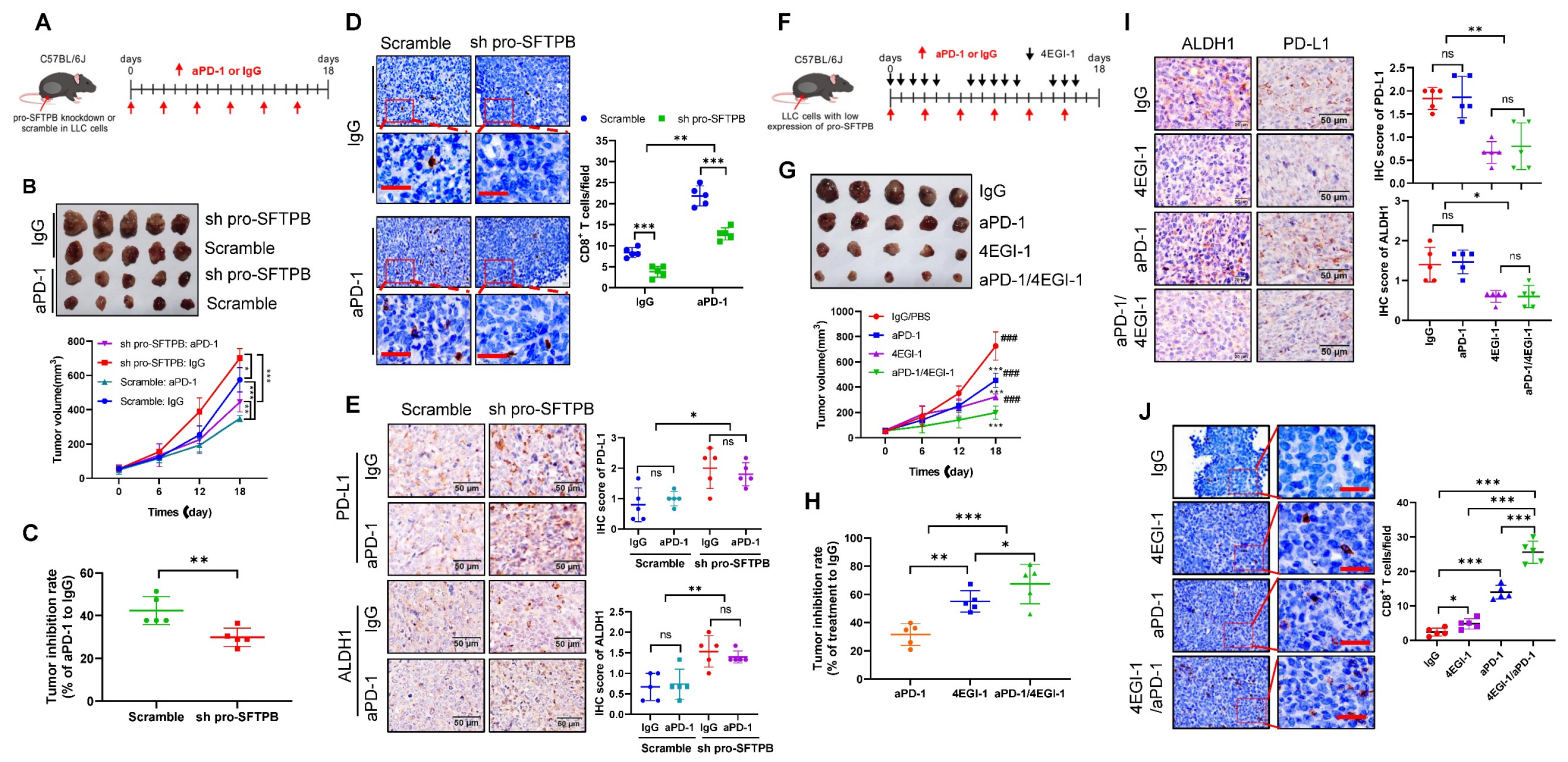
** Knockdown of pro-SFTPB induced resistance to PD-1 monoclonal antibody (aPD-1) in Lewis lung cancer (LLC) cell xenograft models. (A)** Schedule of aPD-1 treatment in C57BL/6J xenograft model constructed by LLC cells transfected with plasmids expressing scramble or pro-SFTPB shRNA.** (B)** Tumor images and growth curve of LLC xenograft tumors that treated with IgG or aPD-1. **(C)** Tumor inhibition rate of aPD-1 treatment on LLC xenograft model with or without pro-SFTPB knockdown (Tumor volume data from B). **(D)** Detection of CD8^+^ T cells by immunohistochemistry (IHC) in LLC xenograft tumors (Tumors from B). **(E)** IHC analysis of PD-L1 and ALDH1 in LLC xenograft tumors (Tumors from B).** (F)** Schedule of indicated drug treatment in C57BL/6J xenograft models constructed by pro-SFTPB knockdown LLC cells. **(G)** Tumor images and growth curve of pro-SFTPB low expressing LLC xenograft tumors that were treated with indicated drugs. *, compared to IgG treatment group;^ #^, compared to aPD-1 treatment group. **(H)** Tumor inhibition rate of aPD-1 or/and 4EGI-1 treatment on LLC xenograft models with low expression of pro-SFTPB (Tumor volume data from G). **(I)** Detection of CD8^+^ T cells by IHC in LLC xenograft tumors (Tumors from G). **(J)** IHC analysis of PD-L1 and ALDH1 in LLC xenograft tumors (Tumors from G). *S*cale bar=50 μm; sh pro-SFTPB, pro-SFTPB shRNA; ns, no significance; *, *p* <0.05; **, *p* <0.01; ***, *p* <0.001; ^###^, *p* <0.001.

The expression levels of c-myc 31 and PD-L1 32 are related to the sensitivity of cancers to PD-1 inhibitors therapy. Thus, we used a C57BL/6J xenograft model constructed with LLC cells to investigate whether downregulation of pro-SFTPB affects the sensitivity of lung cancer to PD-1 inhibitors (Fig. 6A). As shown in Fig. 6B, compared with IgG treatment, PD-1 monoclonal antibody (aPD-1) treatment significantly inhibited LLC tumor growth in both the scramble and pro-SFTPB knockdown groups compared with the corresponding control groups. However, aPD-1 treatment more significantly inhibited tumor growth (Fig. 6B and C) and increased CD8^+^ T-cell infiltration into tumors (Fig. 6D) in the scramble group than in the pro-SFTPB-knockdown group, indicating that downregulation of pro-SFTPB makes LLC tumors resistant to PD-1 inhibitor therapy. In addition, compared with the scramble group, the pro-SFTPB-knockdown group with aPD-1 treatment had higher expression levels of ALDH1 (Fig. 6E). Next, we investigated the effects of 4EGI-1, which blocks eIF4F complex formation, on the efficacy of aPD-1 therapy in LLC tumors with low expression of pro-SFTPB (Fig. 6F). Compared with aPD-1 monotherapy, combination treatment with 4EGI-1 and aPD-1 in LLC tumors with low expression of pro-SFTPB more significantly inhibited LLC tumor growth (Fig. 6G and H) and ALDH1 expression (Fig. 6I) and promoted CD8^+^ T-cell infiltration in LLC tumor tissue (Fig. 6J). Together, these findings suggest that low expression of pro-SFTPB makes LLC cells resistant to PD-1 inhibitor therapy and that blocking the formation of the eIF4F complex can increase the sensitivity of LLC cells with low expression of pro-SFTPB to PD-1 inhibitors.

### Low expression of pro-SFTPB in tumors compared with that in adjacent tissues indicated poor prognosis in rNSCLC patients

Furthermore, by analyzing clinical samples, we investigated whether the molecular mechanism of pro-SFTPB, which has been demonstrated *in vitro* and *in vivo*, exists in rNSCLC patients. Clinical data analysis revealed that 37% of early-stage NSCLC patients (stage Ι and Ⅱ) in our clinical cohort, 34% of early-stage NSCLC patients in the TCGA cohort, and 43% of early-stage NSCLC patients in the GEO cohort had lower expression of pro-SFTPB in tumors than in adjacent tissues (Fig.S5A). In addition, IHC analysis of our clinical cohort samples revealed that early-stage NSCLC tissues with low expression of pro-SFTPB exhibited high expression of PD-L1, c-myc, CD133, and ALDH1 (Fig. S5B and 5C) and reduced infiltration of CD8^+^ T cells (Fig. S5D). In addition, data analysis of stage Ι and stage II NSCLC patients in our clinical cohort (Fig. S5E and S5F) as well as in the TCGA (Fig. S5G and S5H) and GEO (Fig. S5I and S5J) cohorts revealed that low expression levels of pro-SFTPB in tumors compared with those in adjacent tissues were closely related to a lower recurrence-free survival (RFS) rate and a lower overall survival rate in patients with stage Ι and stage II NSCLC.

**Figure 7:**
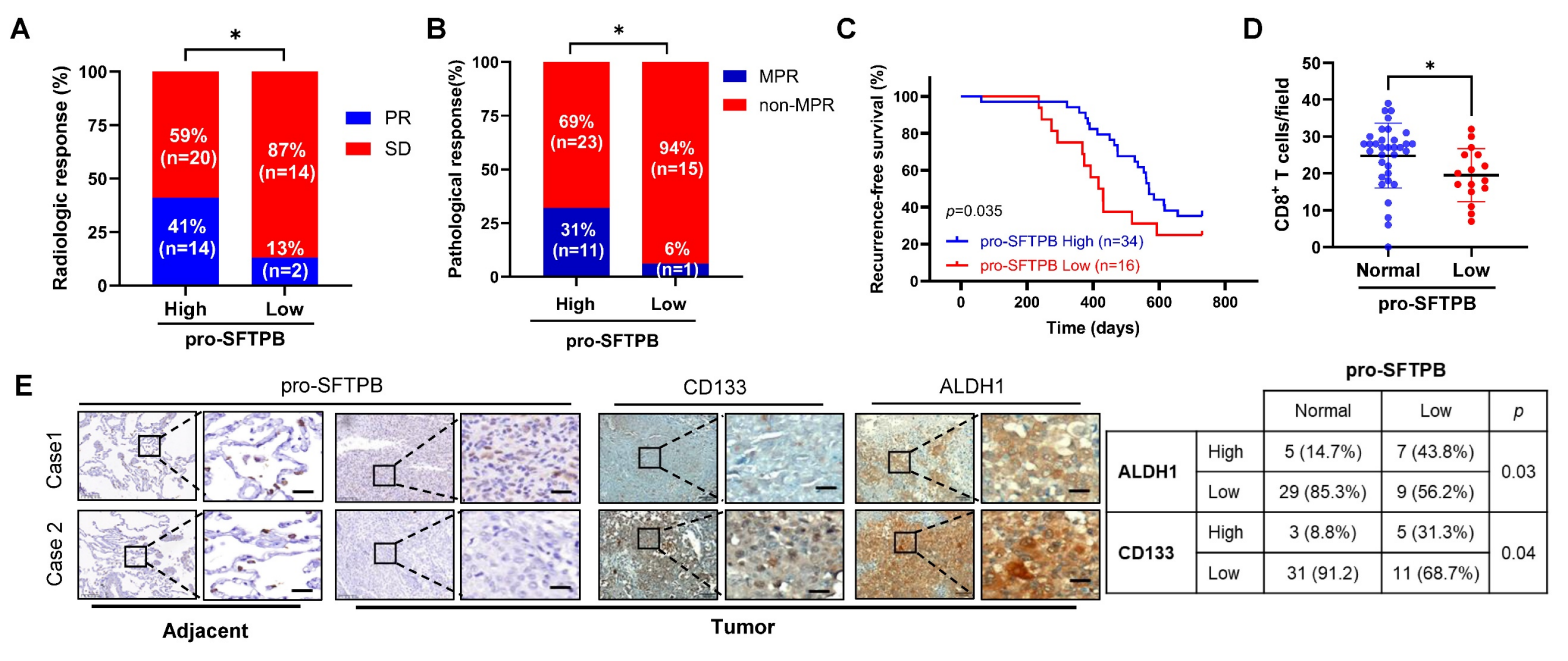
** Low expression of pro-SFTPB in tumor tissues compared to in adjacent tissues is associated with postoperative recurrence in stage IIIA LUAD patients who received neoadjuvant chemoimmunotherapy. (A)** The correlation between pro-SFTPB low expression and radiologic response rate in stage IIIA LUAD patients who have received neoadjuvant chemoimmunotherapy.** (B)** The effect of low expression of pro-SFTPB on the major pathological response of stage IIIA LUAD patients to neoadjuvant chemoimmunotherapy. **(C)** Low expression of pro-SFTPB in tumor tissues compared to adjacent tissues correlated with lower recurrence-free survival rate in stage IIIA LUAD patients who have received neoadjuvant chemo-immunotherapy. **(D)** CD8^+^ T cells were detected in stage IIIA LUAD clinical samples. The significance was calculated by T test. **(E)** Immunohistochemistry (IHC) images of pro-SFTPB, ALDH1 and CD133 in stage IIIA LUAD samples who received chemoimmunotherapy. The significance was examined using Chi-square test. Scale bar=20 μm. PR, partial response; SD, stable disease; MPR, major pathological response. *, *p* < 0.05.

We subsequently investigated the correlation between low expression of pro-SFTPB and the efficacy of neoadjuvant chemoimmunotherapy in LUAD patients with stage IIIA disease. Our clinical data revealed that among stage IIIA LUAD patients who received chemoimmunotherapy (PD-1 inhibitor pembrolizumab/pemetrexed/cisplatin), those with low expression of pro-SFTPB had lower radiological (Fig. 7A) and major pathological responses to chemoimmunotherapy (Fig. 7B) and shorter RFS times (Fig. 7C). In addition, low expression level of pro-SFTPB was negatively correlated with CD8^+^ T-cell infiltration (Fig. 7D) and cancer stemness (Fig. 7E) in stage IIIA LUAD patients who received neoadjuvant chemoimmunotherapy. Collectively, these data suggest that low expression of pro-SFTPB in tumors compared with adjacent tissues is important for poor prognosis and resistance to neoadjuvant chemoimmunotherapy in rNSCLC patients and is closely correlated with increased cancer stemness and immune evasion.

### SFTPB gene transcription was inhibited by smoking, and pro-SFTPB protein expression was positively regulated by the eIF4F complex

**Figure 8:**
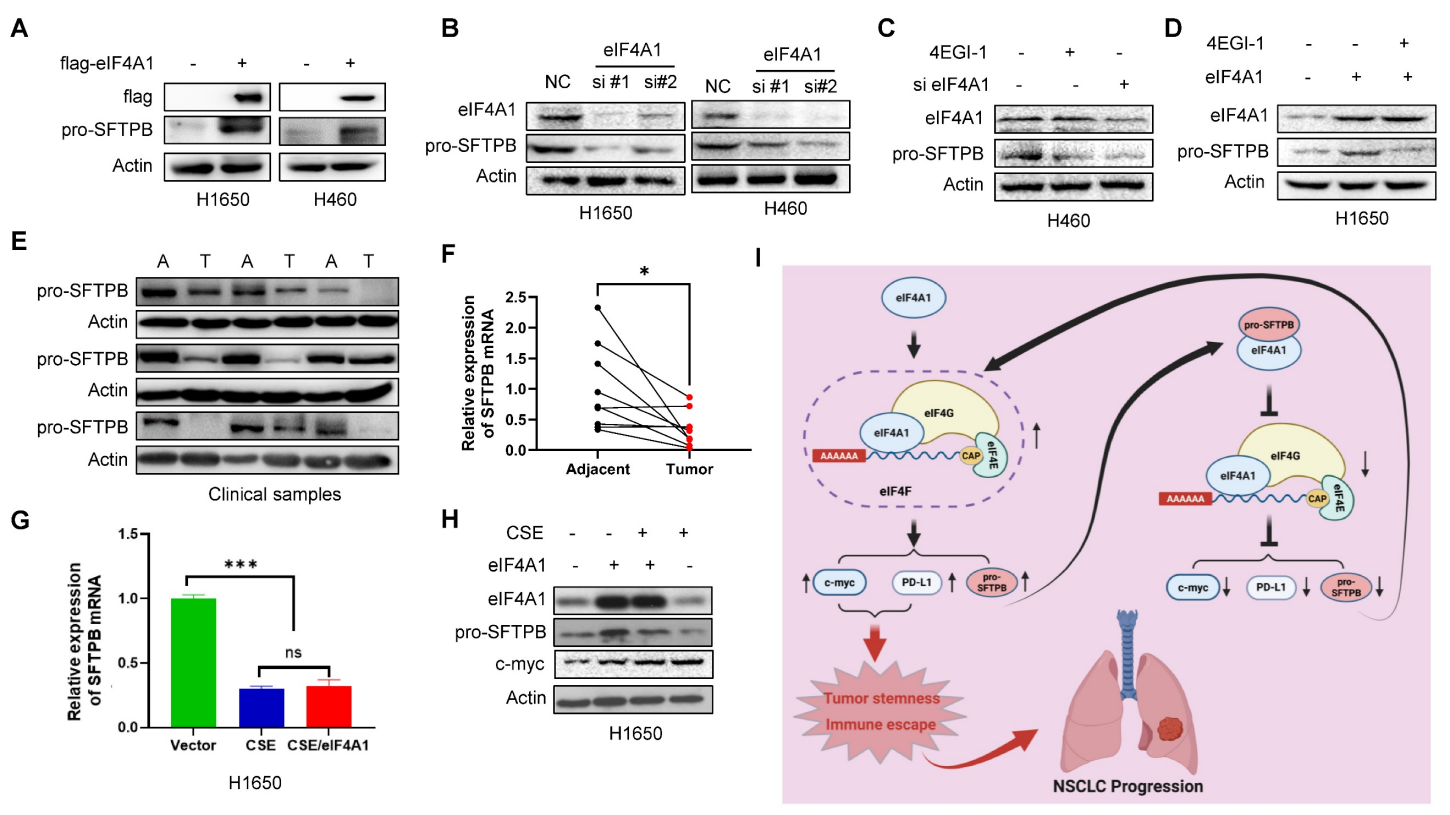
** Smoking reduce SFTPB mRNA and eIF4F upregulates pro-SFTPB protein in NSCLC cells. (A)** Overexpression of eIF4A1 upregulated pro-SFTPB protein level in NSCLC cells. **(B)** Knockdown of eIF4A1 downregulated pro-SFTPB protein level in NSCLC cells. **(C)** Small molecule 4EGI-1 treatment downregulated pro-SFTPB protein level in NSCLC cells. **(D)** Small molecule 4EGI-1 treatment inhibited overexpression of eIF4A1-induced upregulation of pro-SFTPB protein in NSCLC cells. **(E)** The expression levels of pro-SFTPB protein and **(F)** SFTPB mRNA were measured in early-stage NSCLC tissues and their corresponding adjacent tissues (n=9). A, adjacent tissue; T, Tumor tissue. **(G)** Cigarette smoke extract (CSE) inhibited SFTPB mRNA expression levels and **(H)** pro-SFTPB protein levels in H1650 cells. **(I)** The working model of pro-SFTPB in NSCLC progression. *, *p* <0.05; ***, *p*<0.001; ns, no significant.

Finally, we investigated the regulatory mechanism of pro-SFTPB in NSCLC. Our data revealed that overexpression of eIF4A1 increased pro-SFTPB protein levels in NSCLC cells (Fig. 8A), whereas eIF4A1 knockdown reduced the pro-SFTPB protein level in NSCLC cells (Fig. 8B). Importantly, blocking eIF4F complex formation with the small molecule 4EGI-1 dramatically decreased the protein level of pro-SFTPB in NSCLC (Fig. 8C). In addition, 4EGI-1 treatment inhibited the eIF4A1-induced upregulation of pro-SFTPB protein expression in NSCLC cells (Fig. 8D), indicating that SFTPB mRNA translation is positively regulated by eIF4F. To investigate whether the low expression of pro-SFTPB protein in tumors due to the low expression of SFTPB mRNA, we next examined the expression levels of SFTPB mRNA in clinical samples with low expression of pro-SFTPB protein (Fig. 8E). Our results demonstrated that the expression of SFTPB mRNA was significantly downregulated in NSCLC tissues relative to their corresponding adjacent tissues (Fig. 8F). Because clinical data revealed that low expression of pro-SFTPB was correlated with smoking (Table 1), we examined the effects of cigarette smoke extract (CSE) on pro-SFTPB expression in NSCLC cells. The results revealed that CSE treatment significantly inhibited SFTPB mRNA expression (Fig. 8G) and pro-SFTPB protein levels (Fig. 8H) in NSCLC cells. Even when eIF4F-mediated translation was stimulated by eIF4A1 overexpression, the inhibitory effect of CSE on pro-SFTPB protein expression was not affected (Fig. 8G, 8H). Collectively, these findings demonstrate that eIF4F promotes SFTPB mRNA translation, thereby upregulating pro-SFTPB protein levels. However, SFTPB mRNA downregulation in NSCLC by smoking caused abnormal low expression of pro-SFTPB protein.

## Discussion

In newly diagnosed NSCLC, stage Ι to IIIA resectable disease accounts for 45% of all cases, and recurrence is a major challenge in the management of rNSCLC 33. Clinical data have shown that the 5-year recurrence-free survival rates for NSCLC patients with stage Ι, stage II and stage IIIA disease are 68%, 48%, and 20%, respectively 34. Unfortunately, there are currently no biomarkers to predict postoperative recurrence of rNSCLC, and the factors underlying postoperative recurrence are unclear. In this study, by analyzing clinical cohorts from the PLA General Hospital (stages Ι to IIIA) and the TCGA and GEO databases (stages Ι-II), we revealed that approximately 34-43% of rNSCLC patients had decreased expression of pro-SFTPB in tumors compared with adjacent tissues and that low expression of pro-SFTPB was associated with postoperative recurrence of rNSCLC. In addition, a series of *in vitro* and animal experiments have demonstrated that the knockdown of pro-SFTPB significantly promotes the progression of NSCLC. These findings suggest that the downregulation of pro-SFTPB in rNSCLC is an important factor contributing to postoperative progression and has the potential to serve as a predictor of postoperative recurrence.

In the past few years, the treatment paradigm for rNSCLC has shifted as immune checkpoint inhibitors (ICIs) have moved into the early disease stage 35. The US Food and Drug Administration and European Medicines Agency have approved a neoadjuvant ICI for rNSCLC 3. According to the report by Forde et al., compared with neoadjuvant chemotherapy, a neoadjuvant PD-1 inhibitor plus chemotherapy improved the 2-year progression-free survival rate of patients with rNSCLC (stage IB to IIIA) by 18% 36. However, clinical trials have shown that approximately 40% of rNSCLC patients who receive neoadjuvant PD-1inhibitors plus chemotherapy still experience disease progression within 2 to 3 years after surgery 36, 37, indicating that not all patients with rNSCLC benefit from neoadjuvant chemoimmunotherapy. Our clinical data revealed that among stage IIIA LUAD patients who received neoadjuvant PD-1 inhibitor plus chemotherapy, patients with low expression of pro-SFTPB have shorter RFS times and lower major pathological responses to neoadjuvant PD-1 inhibitor plus chemotherapy. In addition, animal experiments demonstrated that downregulation of pro-SFTPB expression induces resistance to PD-1 inhibitors in NSCLC. Notably, targeting eIF4F can improve the resistance of NSCLC with low expression of pro-SFTPB to PD-1 inhibitors. Together, low expression of pro-SFTPB is a factor that induces resistance to PD-1 inhibitors in NSCLC, and targeting eIF4F is a strategy to improve the sensitivity of this type of NSCLC to PD-1 inhibitors.

Next, we elucidated the molecular mechanism through which low expression of pro-SFTPB induces immunotherapy resistance and promotes disease progression in rNSCLC. Our data clearly demonstrated that pro-SFTPB inhibits the formation of eIF4F complexes by binding to eIF4A1, thereby suppressing the translation of genes such as c-myc and PD-L1, which are regulated by eIF4F, thereby suppressing tumor stemness and immune escape 14, 19, 38, 39. Thus, downregulation of pro-SFTPB activates eIF4F regulated cancer stemness and immune evasion, which are important factors in promoting cancer recurrence and leading to chemotherapy and immunotherapy resistance 8, 40-45. Additionally, the constitutive activation of eIF4F also promote cancer progression through the selective translation of other “eIF4F-sensitive mRNAs”, such as CDK4, BCL-2, and MMP9, which are related to cell cycle progression, resistance to apoptosis, and metastasis 46. Collectively, these findings indicate that low expression of pro-SFTPB activates eIF4F-mediated translation by increasing eIF4F complex formation, thereby promoting NSCLC. To our knowledge, this is the first report revealing that the low expression of pro-SFTPB is an important factor leading to the simultaneous activation of cancer stemness and immune evasion and that pro-SFTPB acts as a negative regulator of the formation of the eIF4F complex in NSCLC.

Finally, our study elucidated a regulatory loop between eIF4F and pro-SFTPB protein to coordinate mRNA translation initiation. Our data show that eIF4F mediated translation activation leads to increased expression of pro-SFTPB protein by promoting translation of SFTPB mRNA. pro-SFTPB, in turn, function as a feedback inhibitor of eIF4F complex to inhibit its formation and thus repress eIF4F complex-mediated translation initiation. When eIF4F complex-mediated translation is repressed, the level of pro-SFTPB protein decreases, thereby relieving the inhibitory effect of pro-SFTPB on eIF4F complex formation, ultimately leading to the initiation of eIF4F complex-mediated translation. However, factors such as smoking and inflammation may disrupt the feedback loop between eIF4F and pro-SFTPB in NSCLC by decreasing SFTPB mRNA levels. Our clinical data indicated that low expression of pro-SFTPB is correlated with smoking in rNSCLC patients, and the results of *in vitro* experiments revealed that CSE treatment significantly inhibits SFTPB mRNA expression levels in NSCLC. In addition, Wang et al. demonstrated that treatment with CSE or the proinflammatory factor lipopolysaccharide (LPS) can significantly decrease the mRNA expression levels of SFTPB in NSCLC cells 26. In the deficiency of SFTPB mRNA, even activation of eIF4F-mediated translation cannot increase the protein expression of pro-SFTPB, resulting in the disappearance of the inhibitory mechanism of pro-SFTPB on eIF4F complex formation, ultimately leading to abnormal activation of the eIF4F-meidiated translation pathway.

## Conclusion

In summary, the pro-SFTPB serves as a negative regulator of eIF4F complex formation and plays a role in switching eIF4F-mediated translation. However, the downregulation of SFTPB mRNA caused by smoking in rNSCLC leads to the loss of the inhibitory effect of pro-SFTPB on eIF4F mediated translation by reducing pro-SFTPB protein levels, resulting increased expression of PD-L1 and c-myc regulated by eIF4F, ultimately leading to chemoimmunotherapy resistance and postoperative progression in rNSCLC. Therefore, targeting eIF4F is a strategy to restore the sensitivity of rNSCLC with low expression of pro-SFTPB to neoadjuvant chemoimmunotherapy.

## Supplementary Material

Supplementary figures and tables.

## Figures and Tables

**Table 1:** Characteristics of stage IIIA lung adenocarcinoma patients

Characteristic	pro-SFTPB levels		*p*-value
	High (n=34)	Low (n=16)	
Sex			0.80
Male	14 (41.2%)	6 (37.5%)	
Female	20 (58.8%)	10 (62.5%)	
Age			0.32
60≥	23 (67.6%)	13 (81.3%)	
60<	11 (32.4%)	3 (18.7%)	
Smoking			0.04
Current/former	9 (26.5%)	9 (56.3%)	
Never	25 (73.5%)	7 (43.7%)	
Nodal stage			0.51
N1	4 (11.8%)	1 (6.3%)	
N2	30 (88.2%)	16 (93.7%)	
Tumor stage			0.91
T2	25 (73.5%)	12 (75.0%)	
T3	9 (26.5%)	4 (25.0%)	
PD-L1 levels (TC)			0.04
1-10%	14 (41.2%)	4 (25.0%)	
10-20%	16 (47.1%)	5 (31.3%)	
≥ 20%	4 (11.7%)	7 (43.7%)	

## Data Availability

All data generated during this study are included in this published article and its additional information files.
