# Supplementary material for: Surfactant Protein B Precursor Inhibit the Progression of Resectable Non-small Cell Lung Cancer by Suppressing eIF4F-mediated Immune Evasion and Cancer Stemness
Source: Int J Biol Sci. 2026 Apr 23;22(9):4691–704. doi: 10.7150/ijbs.134006 (PMC13182239; doi:10.7150/ijbs.134006)
Supplement: Supplementary file 1 — Supplementary figures and tables. [file ijbsv22p4691s1.pdf]

Supplementary information

Table S1. Characteristics of patients with early-stage NSCLC (stage I-II)

	Pro-SFTPB expression		*P*
	Normal（n=72）	Low（n=57）	
Sex			0.6205
Male	36	31	
Female	36	26	
Age			0.5937
60≤	37	27	
＜60	35	30	
Smoking			0.3387
Never	50	35	
Current/former	22	22	
Stage			0.1840
I	67	49	
II	5	8	

Table S2. Target sequences of shRNAs and siRNAs

Gene	Sequences
pro-SFTPB Scramble	5'-GCTTCGCGCCGTAGTCTTA-3'
	5'-TAAGACTACGGCGCGAAGC-3'
pro-SFTPB shRNA#1	5'-GATCAAGCGGATCCAAGCCAT-3'
	5'-ATGGCTTGGATCCGCTTGATC-3'
pro-SFTPB shRNA#2	5'-GACTCAAACGGCATCTGTATG-3'
	5'-CATACAGATGCCGTTTGAGTC-3'
pro-SFTPB shRNA#3	5'-GAGGACATCGTCCACATCCTT-3'
	5'-AAGGATGTGGACGATGTCCTC-3'
eIF4A1 siRNA#1	5'-ACCAAGUGCUUGACGACUA-3'
	5'-UAGUCGUCAAGCACUUGGU-3'
eIF4A1 siRNA#2	5'-GACUCAAACGGCAUCUGUA-3'
	5'-UACAGAUGCCGUUUGAGUC-3'

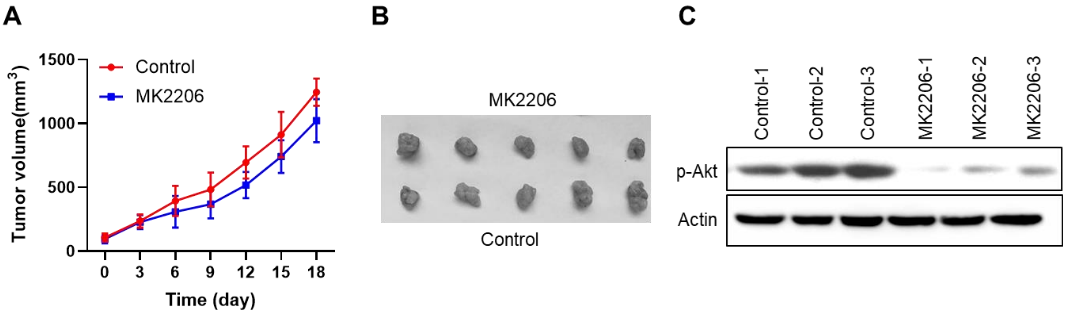

Figure S1. Akt inhibitor treatment did not significantly inhibited tumor growth in C57BL/6J xenograft models constructed using pro-SFTPB silenced Lewis lung cancer cells. (**A**) Tumor growth curve. (**B**) Tumor images. (**C**) p-Akt expression levels in xenograft tumors that from control group and MK2206 treatment group. Xenograft models were constructed using pro-SFTPB silenced Lewis lung cancer cells. When, tumor volumes were reached about 100mm^3, mice were orally administered PBS or MK2206 (50mg/Kg body weight) every three days.

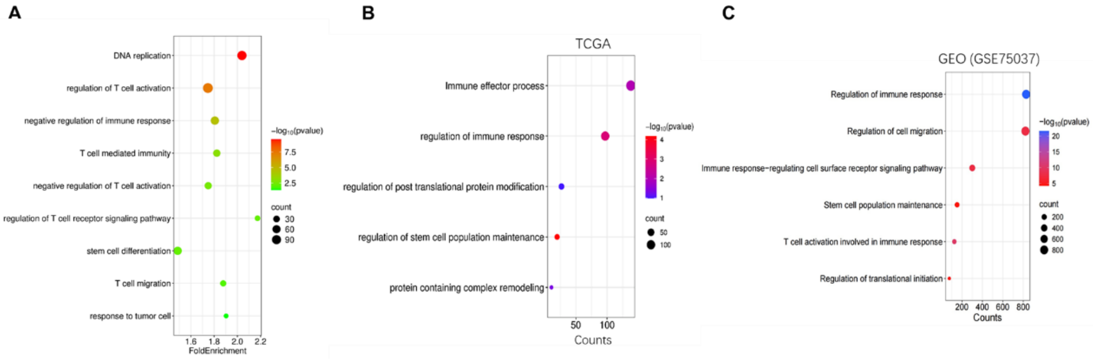

Figure S2. Gene ontology (GO) analysis using early-stage non-small cell lung cancer data. (A-C) GO analysis was performed using sequencing data from our clinical samples, TCGA database, and GEO database.

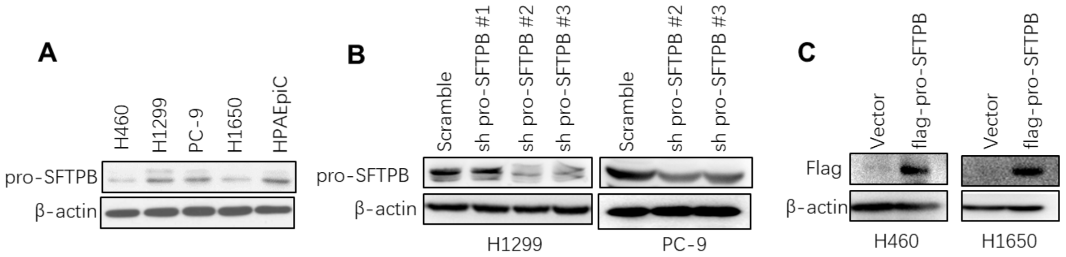

Figure S3. Western blot (WB) analysis of pro-SFTPB in NSCLC cells. (**A**) Detection of pro-SFTPB expression levels by WB in indicated cell lines. (**B**) Indicated cells were transfected with pro-SFTPB shRNA expressing plasmids for 72 hours, then the expression of pro-SFTPB was measured by WB. (**C**) Indicated cells were transfected with pro-SFTPB expressing plasmids for 72 hours, then measured the expression of pro-SFTPB by WB.

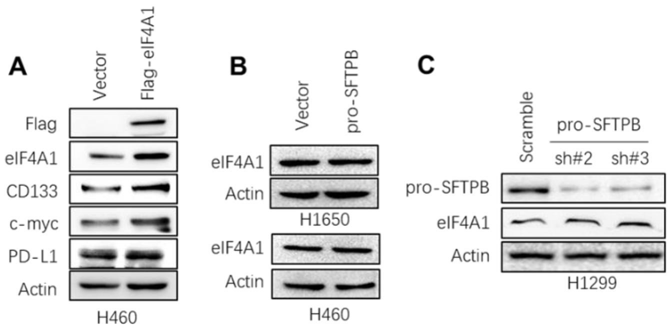

Figure S4. Effect of eIF4A1 on the expression of c-myc and PD-L1, and the effects of pro-SFTPB on the expression of eIF4A1 in NSCLC cells. (A) Western blot (WB) analysis showed eIF4A1 overexpression upregulated c-myc, PD-L1 and CD133 expression in H460 cells. (**B**) WB analysis showed overexpression of pro-SFTPB in H1650 and H460 cells did not change the expression of eIF4A1. (**C**) WB analysis showed knockdown of pro-SFTPB in H1299 cells did not change the expression of eIF4A1. Indicated NSCLC cells were transfected with indicated plasmids for 72 hours, then subjected to WB analysis.

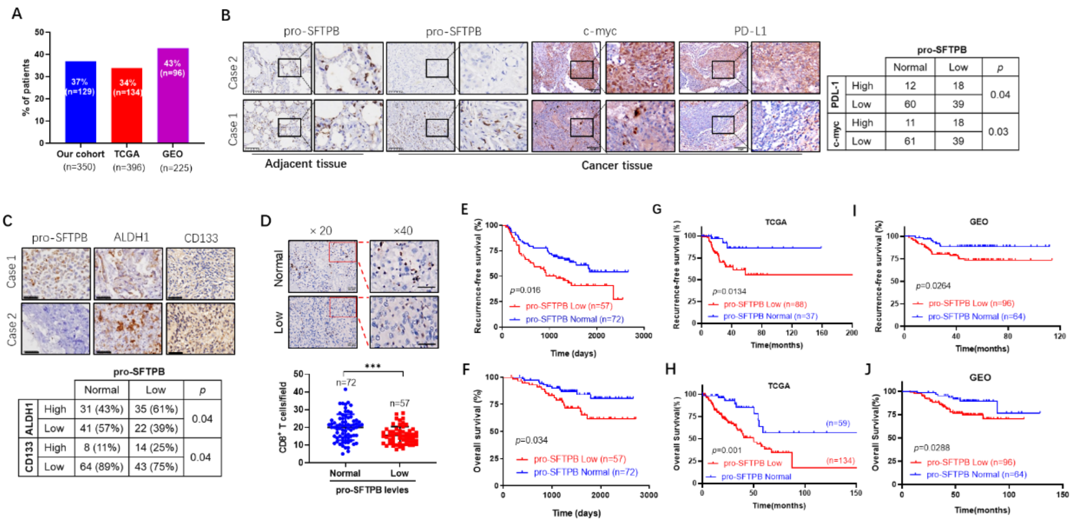

Figure S5. Low expression of pro-SFTPB in tumor tissues compared to in adjacent tissues related to increased cancer stemness, immune evasion, and poor prognosis in NSCLC patients with stage I and II. **(A)** The proportion of early-stage NSCLC patients with low expression of pro-SFTPB in tumors compared to adjacent tissues. **(B)** Immunohistochemistry (IHC) images of pro-SFTPB, c-myc and PD-L1 in early-stage NSCLC tissues. The significance was calculated by Chi-square test. **(C)** IHC images of pro-SFTPB, ALDH1 and CD133 in early-stage NSCLC tissues. The significance was examined using Chi-square test. **(D)** $CD8^+$ T cells in early-stage NSCLC tissues with low or normal expression of pro-SFTPB was detected by IHC. The significance was calculated by T test. **(E-F)** Low expression of pro-SFTPB on NSCLC tissues compared to adjacent tissues correlated with lower recurrence-free survival rate, and lower overall survival rate in early-stage NSCLC patients. **(G-H)** TCGA dataset analysis showed low expression of pro-SFTPB mRNA in NSCLC tissues compared to adjacent tissues correlated with lower recurrence-free survival rate, and lower overall survival rate in early-stage NSCLC patients. **(I-J)** GEO dataset (GSE31210) analysis showed low expression of pro-SFTPB mRNA in NSCLC tissues compared to adjacent tissues correlated with lower recurrence-free survival rate and lower overall survival rate in early-stage NSCLC patients. *S*cale bar=50 μm, ***, p <0.001.
